# Modeling the impact of indoor relative humidity on the infection risk of five respiratory airborne viruses

**DOI:** 10.1038/s41598-022-15703-8

**Published:** 2022-07-07

**Authors:** Amar Aganovic, Yang Bi, Guangyu Cao, Jarek Kurnitski, Pawel Wargocki

**Affiliations:** 1 Department of Automation and Process Engineering, The Arctic University of Norway-UiT, 9019 Tromsø, Norway; 2 Department of Energy and Process Engineering, Norwegian University of Science and Technology-NTNU, 7491 Trondheim, Norway; 3 REHVA Technology and Research Committee, Tallinn University of Technology, 19086 Tallinn, Estonia; 4 Department of Civil Engineering, Technical University of Denmark, 2800 Copenhagen, Kgs Denmark

**Keywords:** Occupational health, Risk factors, Applied mathematics

## Abstract

With a modified version of the Wells-Riley model, we simulated the size distribution and dynamics of five airborne viruses (measles, influenza, SARS-CoV-2, human rhinovirus, and adenovirus) emitted from a speaking person in a typical residential setting over a relative humidity (RH) range of 20–80% and air temperature of 20–25 °C. Besides the size transformation of virus-containing droplets due to evaporation, respiratory absorption, and then removal by gravitational settling, the modified model also considered the removal mechanism by ventilation. The trend and magnitude of RH impact depended on the respiratory virus. For rhinovirus and adenovirus humidifying the indoor air from 20/30 to 50% will be increasing the relative infection risk, however, this relative infection risk increase will be negligible for rhinovirus and weak for adenovirus. Humidification will have a potential benefit in decreasing the infection risk only for influenza when there is a large infection risk decrease for humidifying from 20 to 50%. Regardless of the dry solution composition, humidification will overall increase the infection risk via long-range airborne transmission of SARS-CoV-2. Compared to humidification at a constant ventilation rate, increasing the ventilation rate to moderate levels 0.5 → 2.0 h^−1^ will have a more beneficial infection risk decrease for all viruses except for influenza. Increasing the ventilation rate from low values of 0.5 h^−1^ to higher levels of 6 h^−1^ will have a dominating effect on reducing the infection risk regardless of virus type.

## Introduction

There is an increasing number of scientific evidence that supports the airborne transmission of respiratory enveloped and non-enveloped viruses such as influenza (IV)^1–6^, measles (MeV)^7–9^, rhinovirus (RhV)^4,10^, human adenovirus (HAdV)^11,12^ and the novel severe acute respiratory syndrome coronavirus (SARS-CoV-2)^13–21^. The World Health Organization (WHO) officially acknowledged the inhalation of virus-laden aerosols as the main transmission mode in spreading COVID-19 in 2021^22^. Airborne transmission refers to the inhalation of virus-laden aerosols that can remain suspended for extended periods and transported > 1 to 2 m away from the infected individual in confined indoor spaces^22,23^. The transport mechanism is affected by surrounding airflow distribution and by the size of the virus-laden aerosol. When released from the respiratory tract (assumed to have 99.5% RH), droplets experience rapid evaporation and shrinkage into smaller aerosols upon encountering the unsaturated ambient atmosphere. The ultimate size of an aerosol depends on ambient humidity, and size determines aerodynamic behavior and whether the droplet will settle to the ground quickly or remain suspended in the air long enough to possibly cause an infection. The viral stability of IV, MeV, RhV, HaDV and SARS-CoV-2 has also been shown to be affected by relative humidity at indoor ambient temperature (18–24 °C)^24–28^. It is therefore important to understand the interacting effects of these factors when assessing infection risk in the indoor environment. The Wells-Riley (WR) model is the classic model to assess indoor airborne infection risk quantitatively and has been used extensively to evaluate the risk of respiratory diseases, such as tuberculosis^29^, measles^30^, influenza^31^, and more recently, SARS-CoV-2^32,33^. The original WR model has also been extended to account for three sink or removal mechanisms: ventilation, gravitational settling, and virus inactivation of the airborne pathogen^34^. However, until recently, the removal terms of gravitational settling and virus inactivation in the original WR model could only be calculated for one specific indoor RH value. The additional effect of different indoor RH values on the viral deactivation and deposition removal mechanisms has been recently incorporated into the well-mixed WR model for airborne infection risk assessment of SARS-CoV-2^35^. The modeling results showed that the relative impact of RH on the infection risk depended on the ventilation rate with outdoor air that does not contain the virus. At a relatively low ventilation rate of 0.5 h^−1^, increasing RH from 37 to 83.5% had a strong impact on the infection risk, while at a rate of 6 *h*^−1^, this change had a considerably lower effect. For moderate levels of RH at 40–60% indoors, the ventilation rate was shown to provide a much higher effect in reducing the airborne levels of SARS-CoV-2 than increasing indoor RH. Other respiratory airborne pathogens than SARS-CoV-2 should be considered and evaluated to gain a more general insight into the effects of indoor RH and ventilation rate on the risk of airborne transmission in shared indoor environments. Consequently, we used a WR model similar to the one used before for SARS-Cov-2^35^ to model the mechanisms by which relative humidity may impact infection risk due to airborne transmission of other common respiratory pathogens such as influenza, measles, rhinovirus, and adenovirus. Because the effect of relative humidity on virus inactivation of the aerosolized virus may be influenced by the composition of the aerosol dry solutes composition^36^, in the present study, we supplemented the existing WR model^35^ to account for the effect of a specific amount of organic and inorganic constituents in the dry mass of the aerosol. In addition, we also expanded the WR model to account for the removal by respiratory tract absorption^37^ and considered the impact of RH on this removal term as well. Using the WR model, we illustrate the comparison of how indoor RH might impact airborne transmission infection risk of various respiratory pathogens by simulating the dynamics of airborne viruses emitted from a speaking person in a typical residential setting over a relative humidity (RH) range of 20–80% at ambient temperatures between 20 and 25 °C. Our model advances a mechanistic understanding of the aerosol transmission route, and the results of this study can complement safety guidelines for mitigating respiratory disease infectivity.

## Results

We evaluated the airborne transmission risk of inhaling virus-laden aerosols generated by an infected person speaking continuously under the following conditions: 40 m^2^ room with two occupants (one infected and one susceptible) distanced by at least 1.5 m, and only droplets less than 5 µm in size before evaporation (dehydrated state) are considered for all cases.

**Figure 1:**
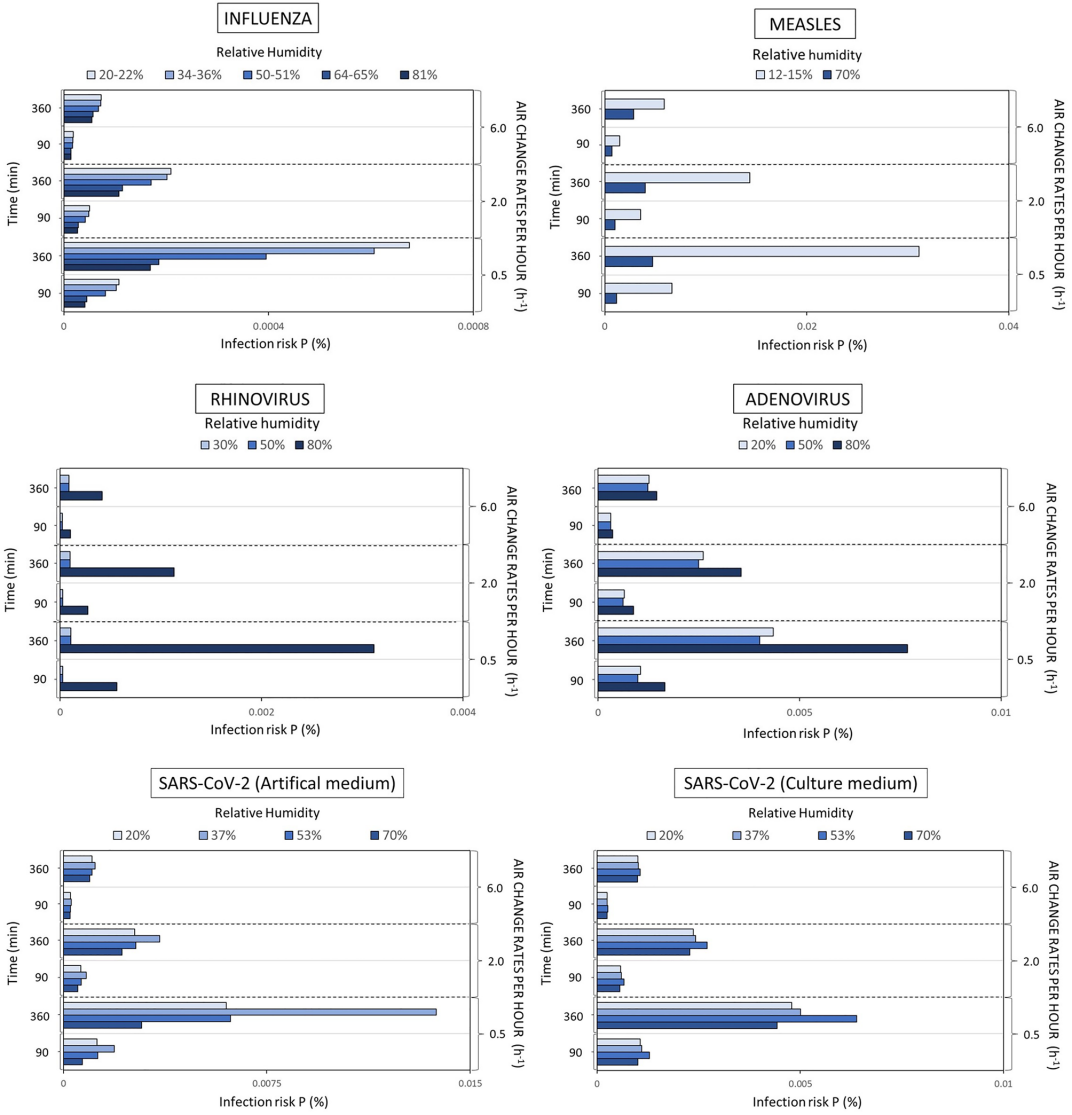
Impact of RH and ventilation rate on infection risk probability P when a person infected with, rhinovirus, adenovirus, SARS-CoV-2 with a dry solute composition consisting of artificial medium and SARS-CoV-2 when the aerosol dry solute composition consists of the culture medium.

The infected person was simulated as an adult male (>18 years old) while the exposed person was simulated as an adult female; both without any pre-existing health conditions. One case was simulated per experiment under

constant breathing rate: 0.54 $\frac{{m}^{3}}{h}$ for the male and 0.49 $\frac{{m}^{3}}{h}$ for the female at standing positions and resting activity. The infected person was assumed to speak for 90 min to simulate short-term exposure risk and 360 min to simulate long-term exposure risk. We considered three ventilation rates, 0.5 h^−1^, which is typical for residential environments in Nordic countries^38^, 2 h^−1^, which can be considered typical for offices and schools with mechanical ventilation^39,40^, and 6 h^−1^, which is recommended for patient rooms by ASHRAE Standard 170 for health care facilities^41^. We have compared our model to two standard models—one which may be interpreted as the conventional Wells-Riley model with only ventilation removal mechanism^42^ and the other being the REHVA (Federation of European Heating, Ventilation, and Air Conditioning Associations) model developed during the pandemics^43^. At a high ventilation rate of 6 h^−1^, there is almost no difference between our model and the two standard models because the ventilation removal mechanism dominates (Supplementary Figs. S1 and S2). However, the model with only a ventilation removal mechanism overestimates the infection risk at 0.5 h^−1^. The REHVA model with both deposition and inactivation removal mechanisms provides more similar results to our model but is still not capable of capturing the RH effect on infection risk. Figure 1 left shows the infection risk probability decreased with higher RH in the case of airborne influenza. The effect of RH depended on the exposure time and ventilation rate—the shorter the exposure time and the higher the ventilation rate, the lower impact of RH on the infection risk. At a ventilation rate of 6 h^−1^, RH’s effect can be considered negligible. Figure 1 shows that the probability of infection risk with measles decreases with increased RH, similarly to Influenza. The impact of RH decreases with increasing ventilation rate but, unlike for influenza, remains relatively strong even at high ventilation rates and especially with increased exposure times. Compared to influenza and measles, the impact of RH on the infection risk of rhinovirus virus is inverse—the probability of infection risk is considerably higher when the relative humidity increases to 80%, compared with 30% and 50% as also shown in Fig. 1. The difference in the probability of infection risk at 30% and 50% is though negligible. The effect of RH remains very strong even when ventilation rates are as high as 6 h^−1^. Figure 1 also shows that increasing RH > 50% increased the probability of infection risk similarly to rhinovirus. Increasing the ventilation rate reduced the effect of RH. The probability of infection risk with adenovirus increases with increased RH, but the difference between infection risks at 80% and 50% is less compared to Rhinovirus. At lower ventilation rates of 0.5 h^−1^ the relative impact of RH becomes weak for adenovirus. We have earlier examined the RH’s effect on the probability of infection risk with SARS-CoV-2^35^, where we averaged the inactivation rates for different aerosol dry solute compositions. Here we examined this effect depending on the distinct saliva/dry solutes composition. The RH on SARS-CoV-2 infection risk is shown in the lower left part of Fig. 1.

For an artificial medium-dry solute composition of 13.1 g/L salts and 3.6 g/L proteins. For this particular composition, the infection risk is non-monotonic and the highest at 37 percent while lowest at 70% RH; there is a negligible difference between infection risks for RH values at 20% and 53%. Increasing ventilation was shown to reduce the effect of RH significantly. In the case of a culture medium-dry solution composition consisting of 17.1 g/L salts and 6.8 g/L proteins, the infection risk is again non-monotonic though the highest at 53 percent; it is the lowest at 70% RH. There is very little difference between the infection risks at 20% and 37% RH. Once the ventilation rate increases, it was observed that RH’s effect would become negligible.

We observed that for non-enveloped viruses such as adenovirus and rhinovirus, increasing RH would increase the probability of infection risk, whereas, for enveloped viruses such as influenza, measles, and SARS-CoV-2, increased humidity will have the reverse effect. This effect will depend on the exposure time and ventilation rates, and the composition of saliva/dry solutes. Increased exposure time will enhance the effect, but increased ventilation will reduce the effect. The exceptions are measles and Rhinovirus, for which the effect of increased RH remains relatively strong even at high ventilation rates. For SARS-CoV-2, the effect of RH is non-monotonic, whereas the medium level of RH increased the risk probability. As the inactivation rates were at least 10 times higher than the deposition rate, the explanation of opposite responses to infection risks can be linked to the presence or absence of a lipid envelope. Enveloped viruses (influenza, measles, and SARS-CoV-2) tend to survive longer at low RH (30%), while non-enveloped viruses (adenoviruses and rhinovirus) survive longer at high RHs (70 to 90%)^44^.

## Practical implications

**Figure 2:**
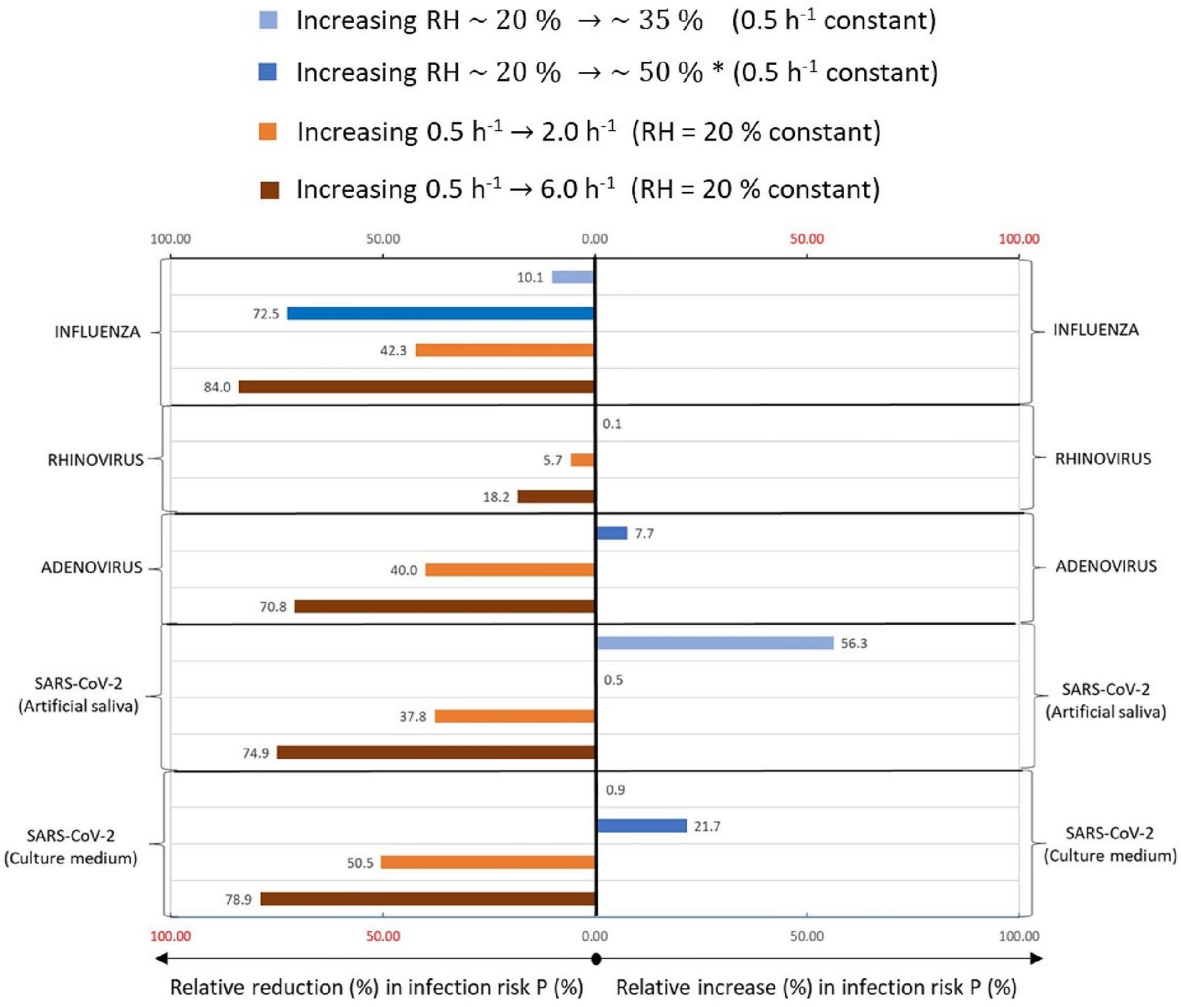
The impact on the relative change in the infection risk of increased humidity from low humidity levels 20% to moderate *≈* 35% and *≈* 50% at constant ventilation rate (0.5 h^−1^) versus the impact of increased ventilation rate from 0.5 to 2 h^−1^ and 6 h^−1^ at constant relative humidity (RH *≈* 20%). *≈ 30% to *≈* 50% for adenovirus.

Present results are discussed in the context of the need for humidification to moderate RH levels of 30–60% in buildings^45^; higher levels are not recommended because of the risks associated with condensation and molds^46^. Figure 2 compares the effect of RH on the different viruses examined in the present analyses by calculating the relative change in infection risk compared with the low RH and low ventilation rates. We present the effect of 360 min exposure when RH increased from *≈* 20 to *≈* 35% and *≈* 50% and compare it with the effect of ventilation increasing from 0.5 to 2 h^−1^ and 6 h^−1^.

Figure 2 shows that for the non-enveloped viruses, humidifying the indoor air by up to *≈* 50% will increase the relative infection risk by 0.1% for rhinovirus and 7.7% for adenovirus. Humidifying will decrease the infection risk only for Influenza. The pattern observed here for Influenza is supported by an experimental study on airborne transmission risk of influenza in mice^47^, where dehumidification showed to increase the infection risk at a constant ventilation rate while increasing ventilation at constant RH decreased the infection risk. Our findings show that the impact of RH is relatively higher when increasing the ventilation rate from 0.5 to to 2 h^−1^ and slightly lower when ventilation is increased to 6 *h*^−1^. For SARS-CoV-2, increasing RH to 50% will generally increase the infection risk; however, this effect will strongly depend on the aerosol dry solution composition (amount of proteins vs. salts). At a higher salt to protein ratio (3.6:1), the impact of increased RH from *≈* 20 to *≈* 35% may increase the relative infection risk more than when RH is increased to 50%. For a lower salt to protein ratio (2.5:1), an increased RH to *≈* 50% will increase the infection risk. Generally, regardless of the dry solution composition, humidification will increase the infection risk via long-range airborne transmission of SARS-CoV-2. Compared to increased RH at a constant ventilation rate, increasing the ventilation rate to 2.0 h^−1^ will considerably decrease the infection risk for all viruses (relative decrease in infection risk is *≈* 38% to *≈* 50% ) except for rhinovirus, where the effect is smaller (5.7% relative decrease). Increasing the ventilation rate to 6 h^−1^ will dominate the reducing infection risk regardless of virus type, ranging from up to *≈* 70% relative decrease for adenovirus *≈* 75–78% for SARS-CoV-2, and up to *≈* 84% for Influenza. Our results are in line with previous studies on influenza reporting that RH > 40% greatly reduces the infectivity of this virus^48–51^. However, the other viruses studied show the opposite effect, increasing RH either will increase infection risk or has no effect. In general, the results in Fig. 2 suggest that humidity will have a different effect on viruses, and there is no optimal humidity level for different viruses. Increased ventilation rate will always reduce infection risk independently of the virus, as supported by scientific evidence^52,53^.

## Conclusion

We showed through modeling that maintaining a higher ventilation rate may have a more beneficial effect on reducing the infection risk of five different airborne viruses than changing RH when considering the virus inactivation, gravitational settling, and respiratory absorption of virus-carrying aerosols. The effect of RH on the human susceptibility to viruses was not considered in this study. Increasing indoor RH from 20/30 to 50% was observed to increase the relative infection risk for SARS-CoV-2, rhinovirus, and adenovirus, but to decrease the infection risk for Influenza. Our results suggest that relative humidity will have a different and even opposite effect on viruses, and there is no optimal humidity level for different viruses, while increased ventilation rate will always reduce infection risk independently of the virus. Public health experts, engineers, and epidemiologists can use the modified model when selecting different measures to reduce the infection risk from various respiratory diseases indoors, allowing informed decisions concerning indoor environmental control.

## Methods

**Figure 3:**
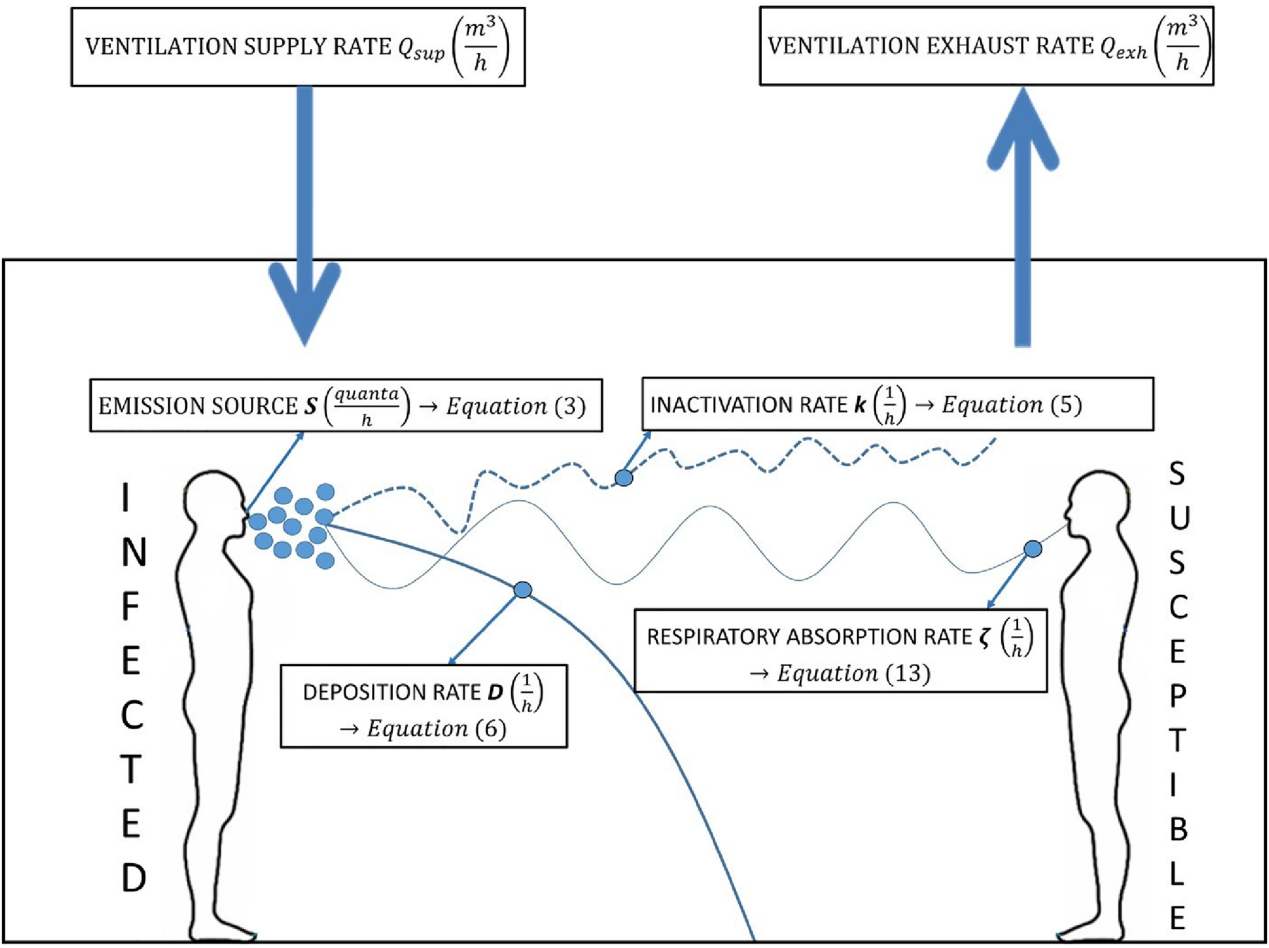
Schematic representation of a simple completely mixed indoor environment including a source term S and removal mechanisms by ventilation, virus inactivation k, deposition by gravitational settling D, and respiratory absorption *ζ*.

A schematic representation of the theoretical model assessing the impact of RH on the virus quanta emission rate for infection risk assessment is shown in Fig. 3. For each of the ventilation models, the following assumptions were made: (i) the only emission source is from the infected individual within the room who emits virus quanta at a constant rate. (ii) there are four removal mechanisms of the infectious quanta: deposition by gravitational settling, virus inactivation by virus inactivation, resuspension, respiratory absorption, and ventilation with recirculation. (iii) indoor airborne transmission is associated with aerosol droplets less or equal than 5 microns in size in the dehydrated state^13^; (iv) only long-range (> 1.5 m) airborne transmission is considered^54^; (v) the volumetric flow rates of air into the room from outside and out of the room to the outside are assumed to be equal and constant for the time interval of the analysis. (vi) Infectious respiratory airborne droplets become evenly distributed throughout each zone considered after reaching steady-state conditions, (vii) no virus-laden airborne particles enter from the outside, (viii) there is no prior source of quanta in the space. (ix) The viral content of a saliva droplet produced by an infected person is proportional to its initial volume^55^. (x) Resuspension rate R is neglected in the model. (xi) There is no simulated sunlight indoors (ultraviolet solar irradiance = 0 $\frac{W}{{m}^{2}}$).

The mass balance model for a non-mixing indoor mechanically ventilated room model can be presented by the following differential equation representing a single-zone model:

1 $$V\cdot \frac{dn\left(t\right)}{dt}=S+{Q}_{sup}\cdot {n}_{sup}\left(t\right)-{Q}_{exh}\cdot {n}_{exh}\left(t\right)-k\cdot n\left(t\right)\cdot V-D\cdot V\cdot n\left(t\right)-R\cdot V\cdot n\left(t\right)-2\cdot \zeta \cdot V\cdot n(t)$$

$V$—the volume of the room,${m}^{3}$; $S$—quanta source emission rate from infected persons (source),$\frac{quanta}{h}$; ${Q}_{out}$—outdoor or supply airflow rate,$\frac{{m}^{3}}{h}$; ${Q}_{sup}$—supply/exhaust airflow rate to/from the room,$\frac{{m}^{3}}{h}$; $n (t)$—quanta concentration in the indoor environment at the time (t),$\frac{quanta}{{m}^{3}}$; ${n}_{sup} (t)$—quanta concentration in the supply/outdoor at the time (t),$\frac{quanta}{{m}^{3}}$; ${n}_{exh} (t)$—quanta concentration in the exhaust at the time (t),$\frac{quanta}{{m}^{3}}$; ${n}_{out}$ = 0—quanta concentration outdoors,$\frac{quanta}{h}$; $k$—virus inactivation rate,$\frac{1}{h}; D$—deposition rate,$\frac{1}{h}$; *R*- resuspension rate,$\frac{1}{h}$; $\zeta$—respiratory tract absorption rate, $\frac{1}{h}$

The unique solution of quanta concentration in an indoor environment with complete mixing ventilation at time t, *n*(*t*) is:

2 $$n(t)={n}_{0}\cdot {e}^{-\left(\frac{ Q}{V}+D+k+2\cdot \zeta \right)\cdot t}+\frac{S}{V}\cdot \left\{\frac{1}{\frac{ Q}{V}+D+k+2\cdot \zeta }-\frac{1}{\frac{ Q}{V}+D+k+2\cdot \zeta }\cdot {e}^{-\left(\frac{ Q}{V}+D+k+2\cdot \zeta \right)\cdot t}\right\}$$

where *n*_0_ is the initial quanta concentration at time t = 0.

To perform calculations with Eq. (2) to predict indoor concentrations of quanta at time t, appropriate expressions for the source term S, deposition rate D, inactivation rate k, and absorption rate *ζ* must first be known.

The pollutant source emission rate S is defined as the virus quanta emission rate generated by infected persons and can be defined by^33^:

3 $$S=I\cdot {c}_{v}\cdot {c}_{i}\cdot IR\cdot \sum_{i=1}^{n}({N}_{i}\cdot {V}_{i})$$

$I$—number of infected persons, -; ${c}_{v}$ —viral load in the sputum, $\frac{RNA}{ml}$ ; ${c}_{i}$—conversion factor is defined as the ratio between one infectious quantum and the infectious dose expressed in RNA copies (quanta/RNA) or tissue culture infectious dose (TCID_50_/ml)*.* The mean values for *c*_*v*_ and *c*_*i*_ are derived from a recent study^56^ (Supplementary Table S2). *IR*—inhalation rate, i.e., the product of breathing (*N*_*br*_) and tidal volume (*V*_*br*_) –are both functions of the activity level of the infected subject. The inhalation rates for resting and standing averaged between males and females are equal to 0.49 and 0.54 $\frac{{m}^{3}}{h}$ , respectively^57^. ${N}_{i}$—droplet number concentration in the ith bin $\frac{particles}{{cm}^{3}}$; ${V}_{i}$ the mean volume of a single droplet (*mL*) in the ith bin:

4 $${V}_{i }\left(D\right)=\frac{\pi \cdot ({{D}_{max}}^{4}-{{D}_{min}}^{4})}{24\cdot ({D}_{max}-{D}_{min})}$$

where *D*_*max*_ and *D*_*min*_ denote the bin’s lower and upper diameter values^58^. ith—size bin of the droplet distribution. The size distribution for talking is determined based on experimental data from two studies measuring the respiratory droplet aerosol distribution for ≤ 2 µm^59^ and for ≥ 2 µm^60^: both studies measured the size distribution of droplets for talking/voice counting at a distance of 10 mm from the participant’s mouth opening (Supplementary Table S1). Therefore, the measured concentration of droplets represents the original size of the droplets at the mouth opening or the mass equivalent diameter of the particle *D*_*eq*_ (m) at the temperature and RH in the respiratory tract (37 °C and RH = 99.5%)^35^. The total volume of droplets was calculated by multiplying the droplet number distribution by the mean volume corresponding to each diameter in the size distribution^35^.

To characterize the impact of relative humidity on the inactivation rate k for the respiratory viruses, data on the aerosolized virus survival times at different relative humidities were obtained from experimental studies^24–28^. The biological decay of virus in aerosols has been reported as the percentage of viable airborne virus (%) measured after continuous time intervals for influenza^24^, measles^25^, rhinovirus^26^ while inactivation rates for adenovirus^27^ and SARS-CoV-2^28^ were reported directly (Supplementary Tables S3–S7).

When a virus is inactivated according to a first-order reaction, the rate of airborne viability *v* (%) can be expressed as:

5 $$\frac{dv}{dt}=-k\cdot v=>ln\frac{v}{{v}_{0}}=- k\cdot t=>k=- \frac{ln\frac{v}{{v}_{0}}}{t}$$

The time t needed for a certain viral loss *v* (%) is found by iterating the estimated regression slopes for each the viability reaches a chosen value *v*. The methodology of calculating *k* from the experimental data available for each specific virus considered is presented in the supplementary data S1.

The deposition rate D of a virus laden-droplet can be expressed as:

6 $$D=\frac{{v}_{s}}{{H}_{person}}$$

${H}_{person}$—the average height of the infected person(s), $m$; The gravitational settling velocity of the droplet ${v}_{s}$, $\frac{m}{s}$; can be determined from:

7 $${v}_{s}=\frac{{C}_{c}\cdot {\rho }_{d}\cdot {Deq}^{2} \cdot \mathrm{g}}{18\cdot \mu }$$

$g$—gravitational acceleration, $\frac{m}{{s}^{2}}$; ${\rho }_{d}$—density of droplets, $\frac{kg}{{m}^{3}}$; ${\uprho }_{\mathrm{a}}$—density of air, $\frac{kg}{{m}^{3}}$; *D*_*eq*_—the droplet equilibrium diameter, $m$.; $\mu$—viscosity of air*,* $\frac{g}{cm\cdot s}$.

${C}_{c}$ is the Cunningham Slip Correction Factor (*−*) and can be determined one of the existing empirical expressions:^61^

8 $${C}_{c}=1+\frac{{{\varvec{\lambda}}}_{g}}{{{\varvec{D}}}_{eq}}\cdot \left(2.51+0.80\cdot {e}^{\frac{-0.55\cdot {{\varvec{D}}}_{eq}}{{{\varvec{\lambda}}}_{g}}}\right)$$

${{\varvec{\lambda}}}_{g}$—mean free path, g/cm^2^.

To perform the numerical iterations for gravitational settling velocity *v*_*s*_ from equation (7) the mass equivalent diameter of the particle *D*_*eq*_ (m) must be known for a specific RH value. In this manner, the mass equivalent diameter of the particle *D*_*eq*_ can be obtained from the Köhler theory^62^ taking into account the two major respiratory fluid components besides water: salts and proteins. The composition of the protein-salt mixture in the aerosol carrier medium across different experiments for deriving inactivation rates is shown in supplementary data (Supplementary Table S8). The inactivation rates for all viruses except for SARS-CoV-2 were reported for one dry solute composition of the aerosol, while the survival rate for SARS-CoV-2 was reported for two compositions: artificial medium culture medium (Supplementary Table S8). Given the dry solutes composition the relationship between the RH and equilibrium droplet diameter *D*_*eq*_ can be derived from the separate solute volume additivity (SS-VA) model for multi-component particles^63^ in the following manner:

9 $$RH=\mathrm{exp} (\frac{4\cdot \sigma {\cdot M}_{w}}{{\rho }_{d}\cdot R\cdot T\cdot {D}_{eq}}-\frac{{M}_{w}}{{\rho }_{w}\cdot \left({\left(\frac{{D}_{eq}}{{D}_{m,s}}\right)}^{3}-1\right)}\cdot \sum_{y}\frac{{v}_{y}\cdot {\Phi }_{y}\cdot {\rho }_{y}\cdot {x}_{s,y}}{{M}_{y}})$$

$$\sigma $$—surface tension of water, $$\frac{N}{m}$$;$$ {M}_{w}$$—molar mass water, $$g/mol$$;$$ {M}_{y}$$—molar mass of dry solutes (protein or salts), $$g/mol$$; $${\rho }_{y}$$—density of dry solutes (protein or salts), $$\frac{kg}{{m}^{3}}$$; $${\rho }_{w}$$—density of water, $$\frac{kg}{{m}^{3}}$$; $$R$$—ideal gas constant, $$\frac{J}{K\cdot mol}$$; $$T-$$absolute temperature, $$K$$; *x*_*s,y*_ is the mass fraction of component *y* in the dry solute, –; *D*_*m,s*_ is the mass equivalent diameter of a particle consisting of dry solutes, $$m$$; $${v}_{y}$$, the stoichiometric dissociation number of the component, –.

${\Phi }_{y},$—the molal or practical osmotic coefficient can be obtained from the following equation:

10 $${\Phi }_{s}=1+\frac{{{g}_{eff,y}}^{-3}\cdot (3-{{g}_{eff,y}}^{-6})}{{(3-{{g}_{eff,y}}^{-3})}^{2}}$$

${g}_{eff,y}$ is regarded as the volume fraction of the pure solute $y$ in the reference solution and is calculated according to:

11 $${g}_{eff,y}={(\frac{{\rho }_{y}}{{\rho }_{s}\cdot {x}_{s,y}}\cdot \left({\left(\frac{{D}_{eq}}{{D}_{m,s}}\right)}^{3}-1\right)+1)}^{1/3}$$

Respiratory tract absorption rate, *ζ,* $\frac{1}{h}$; is a function of droplet diameter and tidal volume size^37^ and can be calculated according to the following equation:

12 $$\zeta =\frac{DE\cdot IR}{V}$$

The total deposition number $DE$ (%) can be approximated by following analytical expression as a function of the equivalent droplet diameter:

13 $$DE\left({D}_{eq}\right)=1-\frac{b}{({De}_{q}\cdot {10}^{-6}+{d}_{0})\cdot \mathrm{ln}(s\cdot \sqrt{2\cdot \pi })}\cdot {e}^{\left[-\frac{1}{2}\cdot {\left\{\frac{\mathrm{ln}\left({De}_{q}\cdot {10}^{-6}+{d}_{0}\right)-\mathrm{ln}{d}_{1}}{\mathrm{ln}s}\right\}}^{2}\right]} $$

where *b* = 5.788, *s* = 2.574*d*_0_ = 1.2 and *d*_1_ = 4.307 are constant values for an average tidal volume of 500 ml which is considered in this study.

To determine the probability of infection (P, %) as a function of the exposure time (t) of susceptible people, the quanta concentration was integrated over time through the Wells–Riley equation as follows:

14 $$P=\left(1-{e}^{-IR{\int }_{0}^{T}n\left(t\right)dt}\right)$$

### Limitations

There are several limitations of our model. First, the modified model was developed for the long-range airborne transmission of SARS-CoV-2, while short-range droplet transmission and direct contact have been confirmed as the significant path to spread the virus, as well did not examine this effect. Second, the impact of human thermal plumes and other heat sources on the flow field has been ignored. Consequently, our method may not be suitable for the scenarios in crowded spaces. Third, the assumption that expelled droplets are evenly distributed in the room air implies an immediate dilution of the expelled virus concentration. In reality, dilution will not occur instantaneously; it highly depends on the movement of the air in the room. Even in a well-mixed room, an exposed person standing directly in front of the infected person may inhale more airborne particles than an exposed person physically distanced at least 1.5 m apart. Another possible limitation is that the resuspension effect has been excluded as a removal mechanism in our version of the Wells-Riley model; previous studies on the airborne transmission of respiratory diseases have shown that disease transmissions could depend on the resuspension of floor dust^64^. The evaluated infection risk does not take into account the reaction of the human immune system to the change of RH. It should also be noted that the infection risks calculated with this model could vary significantly as a function of the activity levels of both the infected subject and the viral load in the sputum of the infected subject. It was also assumed that the infected individual was constantly talking, which may present an unrealistic overproduction of the number of respiratory droplets expelled. Despite the limitation, we believe that the relative effects observed in the present analyses would remain to suggest that increasing RH may not optimal method for reducing the infection risk and that improving ventilation would bring a more considerable impact.

## Supplementary Information

Supplementary Information.
