# Supplementary material for: Modeling the impact of indoor relative humidity on the infection risk of five respiratory airborne viruses
Source: Sci Rep. 2022 Jul 7;12:11481. doi: 10.1038/s41598-022-15703-8 (PMC9261129; doi:10.1038/s41598-022-15703-8)
Supplement: Supplementary file 1 — Supplementary Information. [file 41598_2022_15703_MOESM1_ESM.docx]

Modeling the impact of indoor relative humidity on the infection risk of five airborne respiratory viruses

Amar Aganovic^1*^, Yang Bi^2^, Guangyu Cao^2^, Jarek Kurnitski^3^, Pawel Wargocki^4^

^1^Department of Automation and Process Engineering, UiT The Arctic University of Norway, Tromsø, Norway

^2^ Department of Energy and Process Engineering, Norwegian University of Science and Technology - NTNU, Trondheim, Norway

^3^ REHVA Technology and Research Committee, Tallinn University of Technology, Tallinn, Estonia

^4^ Department of Civil Engineering, Technical University of Denmark, Sweden


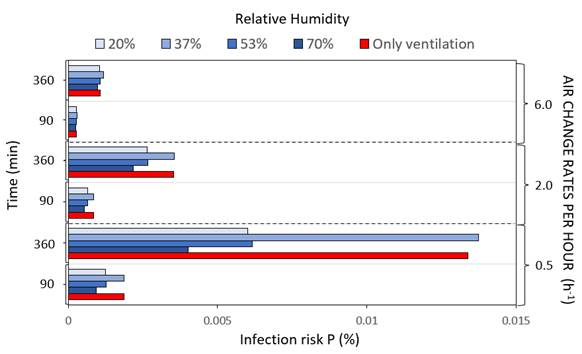


Figure S1. The model developed in this study compared four different RH values for airborne risk infection of SARS-CoV-2 (artificial medium) compared to the conventional Wells-Riley model with only a ventilation removal mechanism.


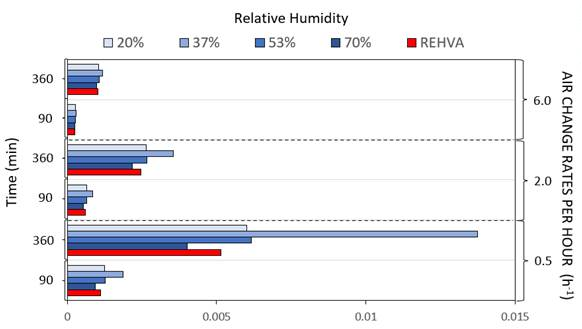


Figure S2. The model developed in this study compared four different RH values for airborne risk infection of SARS-CoV-2 (artificial medium) compared to the REHVA model.

Table S1. Estimated droplet number concentrations $N_{i}$ in different equilibrium diameter $D_{eq}$ ranges emitted during talking/voiced counting and coughing at 10 mm from mouth opening according to Morawska et al. [59] and Chao et al. [60]

| Size range  ($\mu m)$ | Representative droplet diameter at given size range at mouth opening  *D_eq_* ($\mu m)$ | | Calculated mean mass diameter for a dry droplet consisting only of dry solutes  *D_ms_* | $N_{i} (\frac{part}{{cm}^{3}})$  Talking/Voiced counting |
| --- | --- | --- | --- | --- |
| $0.3-0.8$ | | $0.55$ | $0.239$ | $0.118$ |
| $0.8-2$ | | $1.4$ | $0.515$ | $0.152$ |
| $2-4$ | | $3$ | $1.056$ | $0.00459$ |
| $4-8$ | | $6$ | $2.070$ | $0.0662$ |
| $8-16$ | | $12$ | $4.095$ | $0.0223$ |
| $16-24$ | | $20$ | $6.802$ | $0.00113$ |
| $24-32$ | | $28$ | $9.493$ | $0.00787$ |
| $32-40$ | | $36$ | $12.200$ | $0.00432$ |
| $40-50$ | | $45$ | $15.250$ | $0.00447$ |
| $50-75$ | | $62.5$ | $21.181$ | $0.00457$ |
| $75-100$ | | $87.5$ | $29.664$ | $0.00344$ |
| $100-125$ | | $112.5$ | $38.135$ | $0.00452$ |
| $125-150$ | | $137.5$ | $46.662$ | $0.00431$ |
| $150-200$ | | $175$ | $59.321$ | $0.00452$ |
| $200-250$ | | $225$ | $76.115$ | $0.00385$ |
| $250-500$ | | $375$ | $126.864$ | $0.00345$ |
| $500-1000$ | | $750$ | $253.722$ | $0.00111$ |
| $1000-2000$ | | $1500$ | $507.223$ | $0.0000$ |

Table S2. Mean viral load $c_{v}$ and conversion factor $c_{i}$ values for different respiratory viruses [56] carried by droplets $\leq5\mu m$ in dehydrated state

| Respiratory virus | Log_10_ c_v_ mean | Conversion factor c_i_ | quanta/h |
| --- | --- | --- | --- |
| Influenza | 6.7 RNA/ml | 7.1$\cdot10$^-6^ quanta/RNA | $0.166\cdot10$^-3^ |
| Measles | 3.5 TCID_50_/ml | 1.0 quanta/ TCID_50_ | $14.79\cdot10$^-3^ |
| Rhinovirus | 3.6 TCID_50_/ml | 0.053 quanta/ TCID_50_ | $0.99\cdot10$^-3^ |
| Adenovirus | 3.2 TCID_50_/ml | 0.5 quanta/ TCID_50_ | $3.71\cdot10$^-3^ |
| SARS-CoV-2 | 5.6 RNA/ml | 1.4$\cdot10$^-3^ quanta/RNA | $2.6\cdot10$^-3^ |

Virus inactivation rate/biological decay constant$k$

To characterize the impact of relative humidity on the inactivation rate *k* for the respiratory viruses, data on the aerosolized virus survival times at different relative humidities were obtained from experimental studies. The methodology of calculating $k$ from the experimental data available for each specific virus considered is presented in the subsections below.

Influenza

The biological decay of influenza virus in aerosols has been reported as the percentage of viable airborne virus (%) measured after continuous time intervals in a study by Harper [24]. We derived a linear regression slope for each set of measurements at a specific RH correlating the viability (%) as a function of time. When a virus is inactivated according to a first-order reaction, the rate of airborne viability *v* (%) can be expressed as:

$\frac{dv}{dt}=-k\cdot v=>ln\frac{v}{v_{0}}=- k\cdot t=>k=- \frac{ln\frac{v}{v_{0}}}{t}$ (S1)

The time $t$ needed for a certain viral loss *v* (%) is found by iterating the estimated regression slopes in Table 3. for airborne viability of influenza until the viability reaches a chosen value *v.*

Table S3. Calculated regression slopes based on measured airborne viability of influenza at different RH values at 20-24 $℃$ [24]

| Relative humidity (%) | Viability of airborne virus *v* (%) expressed as a function of time *t* (min) | Inactivation rate $k$ (h^-1^) |
| --- | --- | --- |
| 20 – 22 % | $-0.04\cdot t+73.95 (R^{2}=0.89)$ | 0.086 |
| 34 -36 % | $-0.05\cdot t+74.73 (R^{2}=0.77)$ | 0.106 |
| 50 – 51 % | $-0.18\cdot t+60.56 (R^{2}=0.75)$ | 0.485 |
| 64 – 65 % | $0.001\cdot t^{2}-0.49\cdot t+53.84 (R^{2}=0.73)$ | 1.742 |
| 81 % | $0.0011\cdot t^{2}-0.50\cdot t+51.83 (R^{2}=0.75)$ | 1.913 |

Measles

The survival of the measles virus in the air [25] has been reported as the slope coefficient $K$ describing the time-dependent change of the titers of the surviving virus or $\frac{\Delta\log N_{t}}{\Delta t}$ $\left( \frac{\log\left( \frac{plaque-forming units}{ml} \right)}{min} \right)$of the virus in the air which can be rearranged to be expressed as first-order equation (1) for inactivation rate :

$K=\frac{\Delta\log N_{t}}{\Delta t}=\frac{\log N_{t}-logN_{t,0}}{\Delta t}=\frac{\ln\frac{N_{t}}{N_{t,0}}}{\ln10 \cdot\Delta t}$(2) => $k=- K\cdot\ln10$ (S2)

As the slope coefficient has been reported in the original study it is possible to calculate the inactivation rate to reach a specific virus viability *v* 10 % in the same manner as for influenza. The calculated values are shown in Table S4.

Table S4. Calculated regression slopes based on measured airborne viability of measles at different RH values at 20-21 $℃$ [25]

| Relative humidity (%) | Change of titers of surviving virus$\Delta\frac{PFU}{ml}$ expressed as a function of time *t* (min): $\Delta\log N_{t}=K\cdot\Delta t$ | Inactivation rate $k$ (h^-1^) |
| --- | --- | --- |
| 12 – 15 % | $-0.005\cdot\Delta t$ | 0.691 |
| 68 – 70 % | $-0.056\cdot\Delta t$ | 7.736 |

Rhinovirus

The survival of airborne rhinvovirus has been reported in a study by Karim et al. [26] at three different RH values: 30 ± 5 %, 50 ± 5 %, and 80 ± 5 %. The inactivation rate for rhinovirus at RH= 80 ± 5 %. was calculated in the same manner as for influenza based on the regression slope of measured viability at different RH values. At RH = 30 ± 5 % and 50 ± 5 % the viability of the virus was less < 0.25 % after first 15 minutes of measurements. Therefore we calculated the inactivation rate based on a max interval of 15 minutes to reach a viral loss of 99.75 % of the initial value for RH = 30 ± 5 % and 50 ± 5 % (Table S5).

Table S5. Calculated regression slopes based on measured airborne viability of rhinovirus at different RH values at 20-21 $℃$ [26]

| Relative humidity (%) | Viability of airborne virus *v* (%) expressed as a function of time *t* (min) | Inactivation rate $k$ (h^-1^) (h^-1^) |
| --- | --- | --- |
| 30 ± 5 % | < 0.25 % in 15 min | ≤ 23.96 |
| 50 ± 5 % | < 0.25 % in 15 min | ≤ 23.96 |
| 80 ± 5 %. | $-0.043\cdot t+92.93 (R^{2}=0.93)$ | 0.0713 |

Adenovirus

The biological decay rate (% per min) of airborne human adenovirus 7 has been reported in a study by Miller & Artenstein [27] at three different RH values: 20 %, 50 %, and 80 %, as presented in Table S6.

Table S6. Inactivation rates $k$ based on measured airborne viability of adenovirus at different RH values at 20-24 $℃$ [27]

| Relative humidity (%) | Inactivation rate $k$ (h^-1^) |
| --- | --- |
| 20 % | 1.624 |
| 50 % | 1.741 |
| 80 % | 0.492 |

The inactivation rate 𝐾 based on experimental measurements has been reported in a recent study [28]. The reported and calculated values for virus nebulized in artificial saliva, and for virus cultivated in standard tissue culture medium in Table S7.

Table S7. Reported inactivation rates$K$ based on measured airborne viability of Sars-CoV-2 nebulized in artificial saliva at different RH values at 20-20.5 $℃$ [28]

| Relative humidity (%) | Inactivation rate $k$(h^-1^) | |
| --- | --- | --- |
|  | Artificial media | Standard medium |
| 20 % | 0.600 | 0.900 |
| 37 % | -0.150 | 0.780 |
|  |  |  |
| 53 % | 0.480 | 0.450 |
|  |  |  |
| 70 % | 1.050 | 0.900 |

The composition of the protein-salt mixture in the aerosol carrier medium varied across different experiments for deriving inactivation rates as shown in Table S8.

Table S8. Composition of aerosols in experimental studies deriving inactivation rates for different respiratory viruses

| Virus | Salts (g/L) | Proteins (g/L) | |
| --- | --- | --- | --- |
| Influenza [24] | 8.6 | | 2.0 |
| Measles* [25] | n/a | | n/a |
| Rhinovirus [26] | 7.5 | | 20.0 |
| Adenovirus [27] | 0.2 | | 0.9 |
| Sars-CoV-2 ; artificial saliva [28] | 13.1 | | 3.6 |
| Sars-CoV-2 ; culture medium [28] | 17.1 | | 6.8 |

* For measles a simplified model of respiratory fluid composition protein to salt ratio 1:1 was used

The impact of relative humidity on different compositions (protein to salt ratios) in aerosol droplets with an initial size of 5 $\mu$m is shown in Figure S9.
